# Platelet-Derived Extracellular Vesicles as Target of Antiplatelet Agents. What Is the Evidence?

**DOI:** 10.3389/fphar.2019.01256

**Published:** 2019-11-08

**Authors:** Francesco Taus, Alessandra Meneguzzi, Marco Castelli, Pietro Minuz

**Affiliations:** Department of Medicine, Section of Internal Medicine C, University of Verona, Verona, Italy

**Keywords:** platelets, microvesicles, aspirin, thromboxane, P2Y12 inhibitors, atherothrombosis

## Abstract

Platelet-derived large extracellular vesicles (often referred to as microparticles in the field of cardiovascular disease) have been identified as effector in the atherothrombotic process, therefore representing a target of pharmacological intervention of potential interest. Despite that, limited evidence is so far available concerning the effects of antiplatelet agents on the release of platelet-derived extracellular vesicles. In the present narrative review, the mechanisms leading to vesiculation in platelets and the pathophysiological processes implicated will be discussed. This will be followed by a summary of the present evidence concerning the effects of antiplatelet agents under experimental conditions and in clinical settings.

## Biochemical Characterization of Platelet-Derived Extracellular Vesicles

The first evidence of extracellular vesicles (EVs) derived from platelet dates back to 1967, thanks to Wolf (1967), who named them “platelet dusts” even if considered factors promoting clot formation (Nieuwland et al., 2012).

EVs from different cellular origin circulate or are detectable in biological fluids, endowed with different biological functions that are being investigated for their potential pharmacological and clinical relevance. In fact, correlation has been observed between the number of circulating EVs and the clinical expression of diseases such as diabetes mellitus, chronic kidney disease, preeclampsia, and severe hvypertension (Burger et al., 2013; Campello et al., 2015). Thus, platelet-derived extracellular vesicles (PEVs) may represent both useful biomarkers of platelet-mediated pathophysiological processes and mediators of intercellular communication.

Platelets release different types of extracellular products in response to activation and apoptosis. Generally, they are 30- to 800-nm-large vesicles, whose size and amount make them difficult to purify and characterize properly.

The International Society for Extracellular Vesicles (ISEV) proposed the MISEV2018 guidelines, which include suggestions about nomenclature, collection, separation, characterization, and concentrations of EVs (Théry et al., 2018).

ISEV defines EVs as particles released from the cell that are delimited by a lipid bilayer without a functional nucleus. EVs can be classified by physical characteristics such as size (<100-nm small EVs, 100- to 200-nm medium/large EVs, and >200-nm large EVs) or density (low, middle, high, with each range defined) (Théry et al., 2018). ISEV proposes to classify EVs also according to their biochemical characteristics (i.e., CD63^+^/CD81^+^- EVs, annexin A5–stained EVs, etc.) or by descriptions of conditions or cell of origin [podocyte EVs, hypoxic EVs, large oncosomes, apoptotic bodies (ABs)]. Without further classification, the generic term EVs is considered as appropriate (Théry et al., 2018).

EVs, based on their size and supposed origin, can be further grouped into three specific classes: (i) ABs, having an average diameter of 800 to 5,000 nm, which are released by programmed cell death; (ii) large EVs, with a diameter of approximately 50–1,000 nm, deriving from budding of the cell membrane; and (iii) small EVs, also named exosomes, the smallest vesicles of the three classes with a diameter of 40 to 100 nm, released through endocytic process (Kalra et al., 2012; Simpson and Mathivanan, 2012).

Large and small EVs display some specific features that can help to differentiate one from the other. Small EV generation involves tetraspanins (CD9, CD63), tumor susceptibility gene 101 (TSG101), and programmed cell death 6–interacting protein (PDCD6IP or ALIX) (Théry et al., 2018). Furthermore, small and large EVs can be distinguished for their RNA content in terms of RNA types and RNA amount (Tao et al., 2017). Since both large and small EVs carry and deliver cellular signals, it is plausible to suggest a potential role in cell–cell signaling (Dovizio et al., 2015). An important difference between the two families of vesicles is their different biological activity such as the procoagulant activity of large EVs originated from platelets and the involvement of vesiculation in a variety of physiological and pathological processes including angiogenesis, cell proliferation, apoptosis, and inflammation (Heijnen et al., 1999; Tao et al., 2017).

Focus of the present review will be on large PEVs, often named microparticles in the field of cardiovascular medicine, representing 70% to 90% of circulating large EVs in healthy subjects (Horstman and Ahn, 1999;Diamant et al., 2004). Under pathological conditions including cancer, sepsis, diabetes, and acute coronary syndromes, increased number of circulation PEVs has been detected (Shantsila et al., 2010; Varon and Shai, 2015). Deficiency in PEV release is associated with a bleeding disorder characterized by prolonged bleeding time (Castaman et al., 1997). Evidence exists that the number of released PEVs is reduced after pharmacological treatment in cardiovascular disorders (Morel et al., 2006). This has been observed, for instance, in hyperlipidemic patients with type 2 diabetes after treatment with statins and eicosapentaenoic acid (Nomura et al., 2009; Nomura et al., 2018). The effects of antiplatelet agents have so far been addressed only in a limited number of studies, not always as the main endpoint, although this may represent a rational pharmacological intervention aimed at reducing the release of PEVs. For instance, reduced number of circulating EVs from different cellular origin has been observed in patients with acute coronary syndrome treated with aspirin and P2Y_12 _receptor antagonists (Behan et al., 2005; Bulut et al., 2011). However, data are so far controversial, and this is the main topic of the present review that will be discussed in detail further on.

PEVs carry on their surface most of the proteins and receptors that are expressed on platelet plasma membrane. Large PEVs carry the prothrombinase complex and the alpha granule-derived factor V (Sims et al., 1989). Furthermore, large PEVs express receptors including the fibrinogen receptor α_IIb_/β_3_, the von Willebrand factor receptor GPIb, and P-selectin (Mustard et al., 2002). Along with cell-surface proteins, cytosolic content of PEVs includes RNA, miRNA, and perchance DNA that can be transferred to target cells (Burger et al., 2013).

The important role of PEVs in intercellular communication, hemostasis, angiogenesis, and several other physiological and pathological conditions, due to their procoagulant surface, the expression of several receptors, and the cytosolic content, is receiving increasing scientific interest as discussed in several reports (Morel et al., 2008; Vajen et al., 2015; Todorova et al., 2017).

## Isolation, Detection, and Characterization of Platelet-Derived Extracellular Vesicles: Methodological Issues

Different methods are available for PEV isolation, characterization, and quantification, but when multiple parameters have to be considered, the use of different techniques in combination is required (Kailashiya, 2018). In fact, none of the techniques so far used in clinical studies and *in vitro* models allow simultaneous and accurate information on biochemical properties and quantity of PEVs; exhaustive information could only be obtained by combining methodological approaches (Momen-Heravi et al., 2013; Burnouf et al., 2014; van der Pol and Harrison, 2017).

In addition, many preanalytical and postanalytical factors affect PEV measurement and characterization, such as blood collection, handling, and storage (Jy et al., 2004). It is therefore necessary to use any possible precaution and to follow updated recommendations to minimize errors. All these factors have to be taken into account as source of potential bias and limitation in the reliability of experimental studies and clinical trials.

### Isolation and Concentration of Platelet-Derived Extracellular Vesicles

Collection and manipulation of platelets from blood samples require very rigorous handling and processing in order to avoid artifacts and to favor interlaboratory standardizations, first of all using a large needle size (size 21-gauge or larger), discarding the first milliliters of collected blood to prevent platelet activation or desensitization.

The choice of the anticoagulant is important for PEV characterization and quantification, being conditioned by the downstream analysis. The most used anticoagulant is sodium citrate (Robert et al., 2009; Mobarrez et al., 2010; Iversen et al., 2013; Kailashiya, 2018; Zhang et al., 2018; Mitrugno et al., 2019), which minimizes *in vitro* platelet activation and consequent PEV release that is observed with ethylenediaminetetraacetic acid (EDTA) or heparin (Beutler et al., 1990; Gomes et al., 2018). However, EDTA (Chandler, 2016) is suitable when interest is in RNA analysis (Ostenfeld et al., 2016; van Eijndhoven et al., 2016), although affecting EV quantification (Shah et al., 2008; Nelles and Chandler, 2014). Citrate-dextrose solution (ACD) plus EDTA is an alternative anticoagulant for the quantification of circulating PEVs in plasma samples (Shirafuji et al., 2008; Nomura et al., 2009; Shirafuji et al., 2009). ACD alone is used when EVs derived from washed platelets are studied *in vitro* (Castaman et al., 1997; Conde et al., 2005; Pontiggia et al., 2006; Suades et al., 2012; Hsu et al., 2013; Aatonen et al., 2014; György et al., 2014; Vajen et al., 2015; Anene et al., 2018). CTAD (sodium citrate, citric acid, theophylline, adenosine, and dipyridamole) can be useful to prevent *in vitro* platelet activation but can alter intraplatelet signaling, since it increases cytosolic AMP concentration (Mody et al., 1999; Kim et al., 2002). To avoid coagulation of blood or plasma samples, PPACK is used as inhibitor of thrombin-mediated platelet activation, not affecting extracellular calcium concentration (Gemmell et al., 1993; Chandler et al., 2011; Chandler, 2013; Giacomazzi et al., 2016).

Concerning the techniques used in PEV separation and concentration, the MISEV 2018 guidelines define these procedures as follows: separation of EVs (purification or isolation) is generally referred to separation from non-EV component or separation of a specific population of EVs from the other ones. Concentration is the procedure that allows to increase the number of EVs per volume unit with or without separation (Théry et al., 2018).

The most used methods to isolate/concentrate PEVs are based on differential centrifugation (where PEVs can be obtained from supernatant or pellet) or density gradient centrifugation. Isolation depends on the size and mass density or mass density only, but does not separate PEVs from non-EV components such as lipoprotein particles (i.e., chylomicrons), cellular debris, protein aggregates, and very large proteins such as von Willebrand factor (Coumans et al., 2017).

Size exclusion chromatography enables size-based separation on a single column, thus separating EVs from non-EV soluble components (Xu et al., 2016). The choice of matrix determines the size cutoff (e.g., Sepharose 2B has a pore of about 60 nm). Size exclusion chromatography preserves structure and functionality of EVs better than ultracentrifugation (Nordin et al., 2015; Gámez-Valero et al., 2016; Hong et al., 2016). Size exclusion chromatography can be used in association with other techniques such as ultrafiltration in which EVs are retained (Grasso et al., 2015; Nordin et al., 2015).

Immunocapture techniques with monoclonal antibodies and magnetic beads or surfaces are used to isolate subpopulations of EVs on the basis of their immunophenotype (Osumi et al., 2001; Shih et al., 2016; Obeid et al., 2017).

### Detection and Identification of Platelet-Derived Extracellular Vesicles

PEV detection methods can be classified into quantitative (count of EVs) and qualitative (size distribution, morphology, phenotyping, content of proteins o nucleic acid, and functional analysis).

Currently, a single method does not allow phenotyping, sizing, and enumerating PEVs. Standardization of the protocols is therefore recommended to reduce interlaboratory variability (Lacroix et al., 2010a; Lacroix et al., 2010b).

Referring to PEV count, conventional flow cytometry is the mostly used technique. Fluorescein isothiocyanate–phalloidin staining allows to distinguish PEVs from cell fragments in the samples, thus minimizing errors (Lacroix et al., 2010a; Mobarrez et al., 2010; Unsworth et al., 2017). Fluorescent-labeled monoclonal antibodies against CD41 (α_IIb_), CD62P (P-selectin), CD61 (β_3_), and the active fibrinogen receptor α_IIb_/β_3_ (using PAC-1 monoclonal antibody) specifically detect PEVs, besides that most PEVs are also positive for annexin V or lactadherin (Christersson et al., 2010; Connor et al., 2010; Mobarrez et al., 2010; Ayers et al., 2011; Chandler, 2013; Giacomazzi et al., 2016). Annexin V and lactadherin are able to bind phosphatidylserine in a stereospecific manner that is calcium dependent for annexin V and calcium independent for lactadherin (Shi et al., 2003). Unlike PEVs, megakaryocyte-derived large EVs do not express CD62P and LAMP-1 (Flaumenhaft et al., 2009; Italiano et al., 2010; Boilard et al., 2015). Beads are used for the standardization of EV quantification across different cytofluorometric platforms (Robert et al., 2009; Cointe et al., 2016). Standardization is in fact fundamental when the clinical relevance of PEV count is evaluated, allowing multicenter studies.

For *ex vivo* and *in vitro* studies, electron microscopy gives the advantage of detecting very small size particles and is considered the gold standard as imaging technique for PEVs (Brisson et al., 2017). The resolution is in the nanometer scale and allows evaluating structure and morphology of EVs.

Cryo-electron microscopy is useful to study size distribution, morphology, and structure of EVs (Tatischeff et al., 2012).

Atomic force microscopy can be used to count and characterize dimension and distribution of EVs in the nanometer size range (10- to 475-nm range), below the lower limit of detection of conventional flow cytometry. It has been shown that the number of PEVs detected by atomic force microscopy is approximately 1,000‐fold higher than the number detected by flow cytometry (Yuana et al., 2010). Atomic force microscopy can be used to study EVs immobilized on a functionalized surface avoiding interference from abundant proteins (fibrinogen, albumin, immunoglobulins, etc.) and can be coupled to other techniques such as surface plasmon resonance to detect different subpopulations of EVs over a wide concentration range (Gajos et al., 2017; Obeid et al., 2017).

New methodological approaches to the analysis of EVs should allow a more effective characterization of PEVs as summarized in **Table 1**.

**Table 1:** Innovative techniques potentially useful for the detection and quantification of platelet-derived extracellular vesicles (PEVs).

Detection tecniques	Advantages	References
Nanoscale flow cytometry (nFC)	NFC uses high sensitivity multiparametric scattered light and fluorescence measurements, but it needs improvements for intrainstrument and interinstrument standardization and reproducibility. It is used for EV enumeration.	(Gomes et al., 2018)
Imaging flow cytometry (IFCM)	IFCM combines the speed and sample size of traditional flow cytometry with the resolution and sensitivity of microscopy. IFCM has a charge-couple device camera that records both fluorescent intensity and image of the particles, facilitating correct gating and distinguishing EVs from debris swarm detection and other interfering particles.	Erdbrügger et al., 2014; Headland et al., 2014;Erdbrügger and Lannigan, 2016.
Nanoparticle tracking analysis (NTA)	NTA is based on the fluctuation measurement of light scattered by EVs in liquid suspension such as plasma, urine, or washed platelets. NTA allows determination of EV concentration and phenotype when combined with fluorescence (F-NTA) using small sample volume.	Dragovic et al., 2011; Aatonen et al., 2014; van der Pol et al., 2014; Enjeti et al.,2016; Mørk et al., 2016; Rider et al., 2016; Parsons et al., 2017; Ambrose et al., 2018; Nielsen et al., 2019.
Dynamic light scattering (DLS)	DLS is a highly sensitive technique useful for EV counting and size distribution. In this technique, monochromatic light from a laser is directed into a photometric cell containing particles in suspension. Particle sizes (between 1 nm and 6 µm) are determined from fluctuations in scattered light intensity due to the Brownian movement of the particles.	Lawrie et al., 2009; Onódi et al., 2018.
Resistive pulse sensing (RPS)	RPS determines the particle size distribution from resistance pulses caused by particles moving through a pore; this technique is independent of refractive index of the particles tested. The major concerns with RPS are pore clogging and pore stability	van der Pol et al., 2014; Yuana et al., 2015; Eichner et al., 2018
Tunable resistive pulse sensing (TRPS)	TRPS is an adaptation of resistive pulse sensing, in which the pore size can be elastically stretched allowing single particle sizing (until 90 nm) and enumeration in polydisperse sample. TRPS may not be able to discriminate between different types of particles.	Grasso et al., 2015; Maas et al., 2014.
Raman spectroscopic techniques (laser-tweezers Raman spectroscopy, surface enhanced raman scattering)	Used for analysis of biochemical composition of EVs. It has the vantages of quick analysis and it does not require exogenous labeling. It can detect EVs less than 240 nm. The combination of Raman spectroscopy with RPS permits simultaneous information on size, concentration, and chemical composition of single vesicles in suspension without fluorescence antibody labeling.	Lvovich et al., 2010;Tatischeff et al., 2012; Kailashiya et al., 2015.
Time-of-flight secondary ion mass spectrometry (TOF-SIMS)	TOF-SIMS is a spectrometric technique able to examine the chemical detection and molecular compositions of PEVs.	Gajos et al., 2017
Biosensors	Biosensors are new and relatively convenient tools for detection and counting of EVs. Kailashiya et al. developed a graphene oxide-based electrochemical biosensor for detection of pEVs. Graphene oxide-based biosensor seems quick, sensitive, cost-effective, and easy to operate and could be applied at a peripheral health care level as a screening method to identify individuals at high risk of developing coronary artery diseases, which include people having positive family history, history of hypertension, diabetes mellitus, and smoking habits or sedentary lifestyles.	Kailashiya et al., 2015; Obeid et al., 2017

## Platelet Activation and the Release of Large Extracellular Vesicles

Platelet activation in response to soluble agonists (such as thrombin, ADP, and collagen), activators of second messengers (like calcium ionophores and phorbol esters), high shear stress, contact with exogenous surfaces, complement, or low temperatures gives rise to granule secretion and large EV release (Heijnen et al., 1999; Nieuwland et al., 2012). Large PEV formation is an event in which the inner cytoskeleton is disrupted, the symmetry of membrane phospholipids is altered, and the outward plasma membrane undergoes blebbing fragmentation (Morel et al., 2011). Actin filaments, main component of the cytoskeletal, play a major role in PEV formation. The α-actin fibers are cleaved by the Ca^++^dependent protease, calpain, and its inhibition reduces blebbing of PEVs (Yano et al., 1993). Another essential event involved in the release of large PEVs is the externalization of phosphatidylserine, an amino-phospholipid mainly present on the inner leaflet of plasma membrane (Bevers et al., 1999). Flippases promote the translocation of phosphatidylserine and phosphatidylethanolamine toward the inner side of the plasma membrane of the platelet against their own electrochemical gradient through an ATP-dependent mechanism. Floppases, ABC transporter family members (ATP-binding cassette transporter), allow the transport of phosphatidylserine to the outer membrane. Lastly, in an ATP-independent manner, scramblase enzymes help the translocation of phospholipids, inside the two membrane leaflets (Suzuki et al., 2010). Alterations in the membrane phospholipids symmetry and, consequently, the exposure of phosphatidylserine on the plasma membrane turned out to be key mechanisms in PEV generation. It has been shown that lipid-rich microdomains, such as lipid rafts and caveolae, are involved in large EV formation (Biró et al., 2005).

To summarize, remodeling of the cytoskeleton is required for large PEV formation. The reorganization of the cytoskeleton leads to external blebbing of the plasma membrane within lipid-rich microdomains, highly dependent on actin fiber polymerization and their calpain-mediated cleavage (Burger et al., 2013).

Large PEV removal from the circulation has been found to be also caused by phosphatidylserine exposure on the outer membrane leaflet, which not only allows the binding of coagulation factors, but also promotes the elimination of PEVs (Lee et al., 1992; Lemke and Burstyn-Cohen, 2010). Part of PEV clearance takes place in the spleen, so that splenectomized mice show higher levels of CD42^+^ and phosphatidylserine-exposing circulating large PEVs (Dasgupta et al., 2009).

## Platelet-Derived Large Extracellular Vesicles as Mediators in Thrombosis and Hemostasis With Broad Bioactivity

Several studies addressed the mechanism of large PEV generation and their potential bioactivity, with the aim of identifying their biological role and possibly new targets of pharmacological intervention.

PEV generation is dependent on the agonist involved in platelet stimulation (Shai et al., 2012; Milioli et al., 2015; Varon and Shai, 2015). Indeed, the modality of platelet activation is a determinant of size, content, and amount of released PEVs, therefore potentially exerting different effect and having different involvement in the development of diseases associated with platelet activation (Zaldivia et al., 2017).

Large PEVs are important mediators of intracellular communication, transferring their cargo (cytoplasmic or membrane protein, mRNAs, and noncoding RNAs) to target cells (Randriamboavonjy and Fleming, 2018). This explains part of the effects that large PEVs exert in inflammation, thrombosis, immunoregulation, and the transmission of biological information (Boilard et al., 2010). However, the precise mechanism by which PEVs selectively incorporate and release their biological cargo to influence the functionality of target cells needs to be determined (Zaldivia et al., 2017).

Intercellular exchanges of microRNAs mediated by PEVs may modulate gene expression in recipient cells and may determine vascular and tissue response in disease conditions associated with platelet activation (Laffont et al., 2013). Thrombin-activated platelets transfer their miR-223 content to PEVs, which is then delivered to endothelial cells. PEV-derived miR223 regulates the endothelial expression of two of its mRNA target, FBXW7 and EFNA1 (Bartel, 2009; Duchez et al., 2015). High levels of miR-142-3p found in PEVs released by activated platelets, enhance the endothelial cell proliferation/dysfunction via Bcl-2 associated transcription factor (BCLAF)1 (Bao et al., 2018). These results provide a possible mechanism by which activated platelets regulate the function of endothelial cells in hypertension, suggesting a novel potential therapeutic approach based on circulating PEVs.

PEVs may also prove protective in some disease conditions (Berckmans et al., 2001; Owens and Mackman, 2011). PEVs may in fact be involved in remote protection against cardiac ischemia–reperfusion injury (Giricz et al., 2014). Few reports focused on the anticoagulant properties of PEVs. The anticoagulant activity of large PEVs is associated with the binding of the anticoagulant protein S and the activation of protein C. Indeed, protein S specifically binds to PEVs, and the anticoagulant function of the protein C depends on negatively charged surface, therefore acting as potential regulator of the assembly of coagulation factors on PEVs (Dahlbaeck et al., 1992; Somajo et al., 2014). Given the potential procoagulantand anticoagulant effects of large PEVs, the tight regulation of PEV release is likely an important factor regulating the hemostatic process (Zaldivia et al., 2017). Drawing inspiration from structural and mechanistic aspects of the PEVs, Pawlowski et al. (2017) build a liposomal formulation that can be actively anchored to platelet-rich thrombi, releasing encapsulated thrombolytic drugs to generate a site-specific thrombolytic activity. Further molecular and cellular research is warranted to define of the actual effectors of PEVs bioactivity (Ma et al., 2015).

## Antiplatelet Agents and Platelet-Derived Large Extracellular Vesicles: Experimental Studies

PEV formation can be induced *in vitro* by the activation of platelets with agonists, calcium ionophores, phorbol esters, or complement (Sims et al., 1988), but also by a variety of factors including high shear stress (Dachary-Prigent et al., 1995; Holme et al., 1997; Takano et al., 2004), contact with artificial surfaces (Gemmell et al., 1995), and low temperature (Bode and Knupp, 1994). The results from different *in vitro* studies are consistent with a general model in which EVs are released from platelets activated by soluble agonists with the necessary contribution of integrin engagement and shear stress. All these factors are required to generate large PEVs *in vitro* (Giacomazzi et al., 2016). Under these conditions, procoagulant activity is induced, as assessed by measuring the binding of annexin V, index of phosphatidylserine expression, which dramatically increases in PEVs (almost all PEVs become positive) after platelet stimulation (Fox et al., 1991; Barry et al., 1997; Perez-Pujol et al., 2007).

To assess the signaling pathways implicated in large PEV generation, an *in vitro* protocol was set up in our laboratory using antiplatelet agents to investigate the singling pathways implicated in large PEV generation and the potential of antiplatelet agents in reducing their release (Giacomazzi et al., 2016). Platelets generate large PEVs either when stimulated with strong agonists (thrombin, collagen and calcium ionophores), as shown in several independent studies (Fox et al., 1991; Barry et al., 1997; Perez-Pujol et al., 2007), or by weak agonists, such as the thromboxane (TX)A_2_ analog U46619, ADP, and epinephrine (Judge et al., 2010; Zhang et al., 2013). Under these experimental conditions, shear stress needs to be applied to obtain platelet vesiculation (Giacomazzi et al., 2016).

When platelets are activated by soluble agonists in the presence of aspirin or the TXA_2_ receptor (TP) antagonist SQ-29,548, a significant reduction in large PEV release is observed, except when ADP or epinephrine is the stimulus. The inhibitory effects of aspirin and SQ-29,548 indicate a role for endogenous TXA_2_ on agonist-triggered PEV release (Giacomazzi et al., 2016). In the same study, the contribution of ADP secreted from delta granules of activated platelets was assessed using apyrase, an ADP-scavenger enzyme, and PSB-0739, a potent and selective P2Y_12_ receptor antagonist (Hoffmann et al., 2009). Under these conditions, a significant decrease in large PEV released from platelets stimulated with U46619 and low concentration of collagen is observed, not with high-concentration collagen (Giacomazzi et al., 2016). Since similar effects are observed using apyrase and the specific P2Y_12_ inhibitor PSB-0739, it can be inferred that P2Y_12_ is implicated in large PEV generation in response to a large number of agonists. It has also been observed that incubation of normal platelets with aspirin inhibits platelet aggregation and large PEV formation only in response to arachidonic acid, while MeSAMP, a P2Y_12_ inhibitor, blunts platelet aggregation and PEV release induced by arachidonic acid, TRAP, a thrombin PAR-1 receptor agonist, as well as by ADP (Connor et al., 2016). Also, the active metabolite of prasugrel, a P2Y_12_ inhibitor, was shown to strongly inhibit collagen- and TRAP-induced large PEV formation, demonstrating the implication ADP and integrin in this process (Judge et al., 2010).

Therefore, both ADP and TXA_2 _contribute to large PEV release. However, other signaling pathways are implicated in vesiculation, as indicated by the platelet response to epinephrine (Giacomazzi et al., 2016). Large PEV generation does not require platelet secretion, since it has been shown that these two phenomena are dissociated (Delaney et al., 2014). However, large PEV release is entirely dependent on activation of the fibrinogen receptor or an integrin, since eptifibatide, a specific inhibitor of the fibrinogen receptor, prevents the release induced by soluble platelet agonists, except when high-concentration collagen is tested (Giacomazzi et al., 2016). There is evidence that also the engagement of the von Willebrand factor receptor triggers the generation of procoagulant EVs. In fact, the interaction between platelets and immobilized von Willebrand factor leads to the generation of large PEVs under high shear stress (Reininger et al., 2006).

The use of the antiplatelet agents aspirin and a P2Y_12 _in combination, blocking two different but interconnected signaling pathways involved in the amplification phase of platelet activation, determines a more profound inhibition of large PEV release (Judge et al., 2005). Inhibitory activity has been demonstrated also with ticagrelor, a P2Y_12_ antagonist that decreases the release of PEVs exposing P-selectin and procoagulant phosphatidylserine, likely decreasing the proinflammatory/procoagulant interaction between PEVs, monocytes, and coagulation factors (Gasecka et al., 2018). In fact, it has been shown that large PEVs bearing P-selectin, able to bind PSGL-1 on monocytes surface, lead to monocyte activation, responsible for cytokine release and the exposure of tissue factor on monocyte (Falati et al., 2003). Large PEVs exposing phosphatidylserine-bound coagulation factors also propagate thrombin generation (Swieringa et al., 2018).

Glycoprotein α_IIb_/β_3 _inhibitors and P2Y_12_ antagonists are additive to aspirin in preventing large PEV production. Both cangrelor, a P2Y_12_ antagonist, and abciximab, a fibrinogen receptor inhibitor, individually prevented the formation of large PEVs in response to collagen, and the combination of these two agents resulted in further inhibition, while TRAP-induced large PEV formation was insensitive to the effects of aspirin, was reduced by P2Y_12_ or fibrinogen receptor antagonists, and was further inhibited in the presence of both agents (Judge et al., 2005;; Judge et al., 2008).

Additional evidence derives from animal models. For instance, the inhibition of circulating large PEVs by aspirin has been shown to prevent endothelial injury and the progression of early atherosclerotic lesions in experimental diabetes mellitus, suggesting that PEVs may represent a new target of pharmacological intervention in different clinical settings (Wang et al., 2019).

To summarize, *in vitro* studies indicate that the inhibitory effects of antiplatelet agents on PEVs may prove beneficial, beyond inhibition of thrombus formation. All these interactions may potentially contribute to the development and progression of atherosclerosis to atherothrombosis (Badimon et al., 2016). Indeed, PEVs, being involved in cell–cell interaction and transfer of proteins and RNAs to different cells, play a role in a variety of pathological processes including thrombosis and hemostasis, inflammation, atherosclerosis, angiogenesis, and tumor progression (Martinez and Andriantsitohaina, 2011;Owens and Mackman, 2011; Baron et al., 2012; Dovizio et al., 2018). This suggests a broad potential benefit of antiplatelet agents as inhibitors of PEV release.

## Antiplatelet Agents and Platelet-Derived Large Extracellular Vesicles: Clinical Studies

Clinical studies on the impact of antiplatelet agents on PEV release are limited; most of the available data concern large PEVs and derive from nonrandomized clinical trials. In addition, little information is available concerning the relations between PEV generation and clinical outcomes or the relations between pharmacodynamics of antiplatelet agents and PEV release (Rosińska et al., 2017).

Studies concerning treatment with aspirin gave inconsistent results. The generation of procoagulant large PEVs measured in venous blood and in blood taken at the site of a vascular injury in 13 healthy subjects, as assessed by flow cytometry and immunoassays, was not altered after a 7-day treatment with aspirin 100 mg/day in a randomized crossover study (Lubsczyk et al., 2010). It has been demonstrated that patients treated with aspirin undergoing elective coronary artery stenting have high levels of circulating large PEVs, suggesting that aspirin fails to inhibit the PEV formation in that setting (Kim and Kunapuli, 2011). A single study investigated the effects of aspirin given for 7 days to 24 healthy male and female subjects on the release of small PEVs from *ex vivo*–stimulated platelets. No effects of aspirin were observed as for small PEV count, but a significant suppression of their cargo protein levels (Goetzl et al., 2016). The concentration of circulating large PEVs, but not other platelet parameters, was found to be decreased after antiplatelet therapy with aspirin 100 mg/day plus cilostazol 200 mg/day, given for 4 weeks to 112 patients with acute ischemic stroke in a nonrandomized clinical trial including 35 control subjects (Chen et al., 2015). In that study, circulating large PEVs were found to be an independent risk factor for the infarct size in a pooled analysis of patients with ischemic stroke after adjustments of other factors including hypertension and diabetes mellitus (Chen et al., 2015).

In comparison with healthy subjects, patients with stable angina show increased release of PEVs that decreases after administration of low-dose aspirin. When tested *in vitro* in rat thoracic aorta, PEVs collected from patients decreased the tissue expression of ERK1/2 and increased the expression of p38 mitogen-activated protein kinases (MAPKs), c-Jun N-terminal kinases (JNKs), nuclear factor kappa-light-chain-enhancer of activated B cells (NF-kB) and vascular cell adhesion molecule 1 (VCAM-1) and the generation of superoxide anion while decreasing the release of NO. Treatment with aspirin significantly inhibited all these cellular responses, with efficacy similar to that of ERK1/2, p38 MAPKs, and NF-κB inhibitors (Cheng et al., 2017).

Treatment with aspirin (100 mg/day) was shown to decrease the number of circulating endothelial- and platelet-derived large EVs in 15 male patients with coronary artery disease, but nonsignificant coronary artery stenosis, in a nonrandomized clinical study (Bulut et al., 2011).

Furthermore, in 160 male patients with stable coronary heart disease, elevated concentration in peripheral blood of large PEVs bearing tissue factor, together with coexistent hypertriglyceridemia, was among the factors supporting the resistance to the antiplatelet activity of clopidogrel (Jastrzebska et al., 2018).

The inhibitory effects of clopidogrel on large PEVs were found to be related to the maximum plasma concentration reached by clopidogrel active metabolite and the area under the curve observed in a pharmacokinetics/pharmacodynamics study including 26 patients with stable coronary artery disease (França et al., 2012).

Using flow cytometry, the release of large EVs from platelets, monocytes, erythrocytes, and smooth muscle cells was found to be increased in patients with diabetes mellitus. Aspirin therapy reduces biomarkers of vascular wall cell activation and large EV shedding from smooth muscle cells and erythrocytes, but not from platelets, although decreases the number of tissue factor–bearing PEVs with similar effects in 43 patients with types 1 and 2 diabetes mellitus (Chiva-Blanch et al., 2016). Referring to a different clinical setting, recurrent increase in circulating large PEVs was observed after discontinuing aspirin in 17 of 46 patients with Kawasaki disease, a chronic inflammatory arterial disease (Kim et al., 2017).

The additive contribution of platelet P2Y_12_ receptors in the setting of acute vascular disease is well recognized. Dual antiplatelet therapy with aspirin and a P2Y_12 _inhibitor is commonly used in these patients to reduce their very high thrombotic risk (Hechler and Gachet, 2015, Nylander and Schulz, 2016), but the effect of this combination therapy on the release of PEVs has not yet been extensively investigated (Connor et al., 2016). In a randomized clinical trial comparing treatment with aspirin alone or in combination with clopidogrel in 70 patients with ischemic stroke, the addition of aspirin to clopidogrel was found to be associated with significant inhibition of collagen-induced platelet aggregation and diminished formation of platelet–monocyte large EVs (Serebruany et al., 2005). Extended-release dipyridamole plus aspirin, clopidogrel plus aspirin, or clopidogrel alone similarly reduced different markers of platelet activation, while only aspirin plus clopidogrel diminished the formation of platelet–monocyte large EVs in a randomized, single-blind clinical trial including patients with type 2 diabetes mellitus and history of transient ischemic attach (Serebruany et al., 2008).

In conclusion, the hypothesis that PEVs may have a pathogenetic role in atherothrombosis is strong. The secondary hypothesis that modulation of PEV generation may contribute to the antithrombotic activity of antiplatelet agents is so far supported by weak experimental evidence. *In vitro* studies indicate that activation of the arachidonic acid pathway and the release of ADP from delta granules are implicated in the release of large EVs from platelets. Therefore, both aspirin and P2Y_12_ inhibitors may be beneficial also *in vivo* by reducing the prothrombotic potential of activated platelets mediated by released PEVs. Since the release of PEVs is strongly dependent on integrin activation, inhibitors of the fibrinogen receptor, von Willebrand factor receptor, and collagen receptor may prove effective *in vivo*. None of these potential activities have so far been exhaustively demonstrated. A number of clinical trials describe variations in PEV concentration in blood collected from patients treated with antiplatelet agents. However, inconsistency in the study protocols, the small sample size, and differences in the methods used to quantify PEVs represent major limitations. Since PEVs may have a pathogenetic role not only in atherothombosis, but also in other clinical settings, including inflammatory diseases (Boilard et al., 2010) and cancer (Varon and Shai, 2015), broad beneficial effects of antiplatelet intervention may be expected. Further investigation on the efficacy of antiplatelet agents as inhibitors of PEVs with *ad hoc*–designed controlled clinical trials is warranted.

## Author Contributions

All authors listed have made a substantial, direct and intellectual contribution to the work, and approved it for publication.

## Funding

The study was supported by a research grant given by Cariverona Foundation to FT.

## Conflict of Interest

The authors declare that the research was conducted in the absence of any commercial or financial relationships that could be construed as a potential conflict of interest.
